# Biomarkers in Body Fluids as Indicators of Skeletal Maturity: A Systematic Review and Meta-analysis

**DOI:** 10.5041/RMMJ.10506

**Published:** 2023-08-30

**Authors:** Priyanka Kapoor, Rajiv Balachandran, Aman Chowdhry, Giuseppe Perinetti, Om Prakash Kharbanda

**Affiliations:** 1 School of Dental Sciences, Sharda University, Greater Noida, Uttar Pradesh, India; 2 Department of Orthodontics, Faculty of Dentistry, Jamia Millia Islamia, New Delhi, India; 3 Department of Orthodontics and Dentofacial Orthopedics, Centre for Dental Education and Research, All India Institute of Medical Sciences, New Delhi, India; 4 Department of Oral Pathology & Microbiology, Faculty of Dentistry, Jamia Millia Islamia, New Delhi, India; 5 Private Practice, Nocciano (PE), Italy; 6 Faculty of Dental Sciences, M.S. Ramaih University of Applied Sciences, Bangalore, India; 7 Health Sciences, M.S. Ramaih University of Applied Sciences, Bangalore, India

**Keywords:** Biomarkers, CVMI, gingival crevicular fluid (GCF), saliva, serum, skeletal maturation index

## Abstract

**Objectives:**

This review aimed to critically appraise the evidence for biomarkers in blood serum, gingival crevicular fluid (GCF), saliva, and urine in comparison with standard radiographic indices for skeletal maturation assessment.

**Materials and Methods:**

A thorough literature search in multiple databases was conducted for biomarkers in body fluids for skeletal maturation assessed with cervical vertebrae in lateral cephalograms or on hand-wrist radiographs. Different combinations including free text, MeSH terms, and Boolean operators were used. Two researchers used strict inclusion and exclusion criteria to screen title, abstract, and full text, and used the Quality Assessment of Diagnostic Accuracy Studies (QUADAS)-2 instrument for risk of bias assessment of individual studies. Meta-analysis was performed on eligible studies using RevMan 5 software.

**Results:**

A total of 344 articles were screened, of which 33 met the inclusion criteria and quality assessment. The skeletal maturity indicators included insulin-like growth factors (IGF-1), alkaline phosphatase (ALP), bone-specific alkaline phosphatase (BALP), dehydroepiandrosterone sulfate (DHEAS), vitamin D binding protein (DBP), parathormone-related protein (PTHrP), osteocalcin, metalloproteins, and serotransferrin (TF) along with different metabolites. At puberty, a significant rise was seen in IGF-1, DBP, ALP, osteocalcin, TF, and BALP. However, the serum DHEAS and PTHrP increased from pre-pubertal to post-pubertal stages. Due to the data heterogeneity, a meta-analysis could be performed on seven studies in total on IGF-1 in serum and blood. Of these, five were included for data in males and six in females, and four studies on IGF-1 in serum and blood. A significant difference in IGF-1 levels was seen between stages of peak pubertal growth spurt (CS3 and CS4) and decelerating pubertal growth (CS5) compared with growth initiation stage (CS2).

**Conclusions:**

Pubertal growth spurts were correlated with peak serum IGF-1 and BALP in both sexes individually. Peak ALP levels in GCF were correlated with the pubertal spurt in a combined sample of males and females. Standard biofluid collection protocols and homogeneity in sampling and methodology are strongly recommended for future research.

## INTRODUCTION

Skeletal maturity evaluation (SME) is integral to orthodontic diagnosis and treatment planning for a successful outcome. Management of certain skeletal malocclusions including a retrognathic or small mandible can be treated with jaw growth modification. The facial growth modifications are known to perform best at pubertal onset and attaining peak height velocity (PHV). In addition, orthognathic surgery with orthodontics is performed only after cessation of active skeletal growth, for which assessment of growth status becomes extremely important.^1^ Various anthropometric and radiographic methods are used in clinical practice. The radiographic techniques include hand-wrist radiographs based on changes in morphology and ossification of carpal bones, and lateral cephalograms based on cervical vertebral maturation index (CVMI), the staging of which is commonly referred to as cervical stages (CS), and cervical vertebral maturation (CVM) stages.^1^ These have been correlated with dental maturation stages, PHV, and other physical growth parameters in studies for skeletal maturation.^2^–^4^

Todd developed SME based on hand-wrist radiographs, which Greulich and Pyle popularized by creating an atlas.^5^ This method was further developed as a scoring system by Tanner Whitehouse^5^ and as 11 skeletal maturity indicators (SMIs) by Fishman^6^ and further studied solely for the middle phalanx of the third finger (MP3) for skeletal maturation.^7^ The CVMI on lateral cephalograms has been extensively studied for SME,^8^,^9^ primarily in dentofacial disharmonies,^9^–^12^ and is subject to inter-operator variations.^13^ In comparison, the hand-wrist radiographs are still considered more reliable up to the age of 14, although with a risk of additional radiation exposure.^14^

Contemporary orthodontic research is inclined toward biomarkers associated with bone turnover in growth and remodeling.^15^ Although this research has been well established in association with tooth movement,^16^–^18^ the role of such biomarkers in skeletal maturation is yet to be recognized. Evidence supports a rise in the level of bone alkaline phosphatase (BALP) in the serum of pre-pubertal girls, followed by a decrease at puberty and late puberty. 19 Peak serum insulin growth factor-1 (IGF-1) and BALP are seen in pubertal onset, while pubertal stage shows peak serum osteocalcin and type I procollagen peptide.^19^ The biomarkers IGF-1 and BALP have shown a definite relationship with cervical maturation staging.^20^–^23^

While the biomarkers for tooth movement are mainly found in gingival crevicular fluid (GCF),^16^,^17^ the markers for skeletal maturation are generally sourced in blood and serum.^20^–^22^ Certain body fluids are advantageous in terms of non-invasive and repeatable collection, hence the current trend has shifted focus on biomarker research in GCF and saliva where variations in levels of alkaline phosphatase (ALP), vitamin D binding protein (DBP), and serotransferrin (TF) at different stages of skeletal maturation have been reported.^24^,^25^ Levels of ALP have been studied in the saliva of rats for systemic bone turnover^26^ and are currently being explored in human saliva for bone maturation.^27^ A recent scoping review^5^ studied biomarkers of skeletal maturation in saliva and GCF but did not include other body fluids, i.e. blood, serum, and urine, while another recent systematic review^28^ focused only on serum biomarkers in association with radiographic skeletal maturity indicators.

Hence, the current systematic review is aimed at performing a critical appraisal of all available evidence related to biomarkers in different body fluids (blood, GCF, saliva, serum, and urine), compared with established radiographic indices for skeletal maturation, i.e. CVMI, hand-wrist, and middle phalanx of the third finger. We aim to outline the biomarker dynamics in different skeletal maturation stages and explore association(s) of biomarker levels with maxillo-mandibular growth parameters, including average, early, and late maturers, and sex differences.

## MATERIALS AND METHODS

### Protocol and Registration

The current systematic review and meta-analysis were performed after prior registration in PROSPERO (CRD42016049051).

### Eligibility Criteria, PICO

The primary research aim was to study the change in biomarker levels in different body fluids (GCF, saliva, blood, serum, and urine) of orthodontic subjects during different phases of skeletal maturation. The research was framed using the PICO (population, intervention, comparator, outcome) format, as follows: *participants*, orthodontic subjects (both males and females in all age groups); *intervention*, biomarker collection in different body fluids; *comparator*, standardized radiographic indices for skeletal maturity; *outcome*, the primary outcomes were biomarker dynamics in different skeletal maturity stages, and the secondary outcome was variation in biomarker levels with sex or other skeletal parameters like mandibular growth or changes in vertical height. There was no restriction on language or date.

### Information Sources, Search Strategy, and Study Selection

A thorough literature search was conducted, in April 2023, in major databases with a pre-determined search strategy (Supplementary Table 1)—PubMed, Web of Science, Directory of Open Access Journals, Scopus, and Embase, along with a hand search and reference tracking. Two reviewers (PK and RB) independently applied the inclusion and exclusion criteria for studies (Supplementary Table 2). Forty-two studies were shortlisted based on full-text retrieval and an in-depth review. Of these, one study was excluded due to non-availability of a radiographic comparator.^29^ The remaining 41 studies were further assessed for quality with a modified Quality Assessment of Diagnostic Accuracy Studies (QUADAS)-2 instrument.^30^

**Table 1:** Participant and Study Characteristics of Included Studies.

#	Reference	Study Design	Participants	X-ray	Skeletal Maturity Method	Biomarker(s)	Body Fluid	Results
1.	Almalki, 2022^37^	CSS	105 (62 F, 43 M), 6–25 y	LCR	Cervical vertebral maturation stages (CVM stages):^9^ 6 CS	IGF-1, IGFBP-3, IGF-1/ IGFBP-3 ratio	Saliva	Highest mean IGF-1 at peak puberty (CS3 and 4): F=1.57 ng/mL; M=2.57 ng/mL Lowest mean IGF-1 at pre-puberty (CS 1 and 2): F=0.85 ng/mL; M=1.22 ng/mL Significant difference between M and F at puberty (*P*<0.01) but no significant difference in pre- and post-puberty Significant interaction of stages and sex on IGF-1 (*P*<0.05) but not on IGFBP-3 and IGF-1/IGFBP-3 molar ratio (*P*>0.05)
2.	Almalki et al., 2022^38^	CSS	90 (34 M, 56 F), 6–25 y	Hand-wrist	5 SMI stages^36^	IGF-1, IGF-1/IGFBP-3 ratio	Saliva	IGF-1 levels low at pre-puberty, then increase at puberty onset and peak, followed by a decrease at growth deceleration stage to growth completion. Strong positive correlation (*r*=0.98, *P*<0.01) btw IGF/IGFBP-3 ratio and SMI staging
3.	Al Meshari and Aldweesh, 2022^39^	CSS	141 (70 M, 71 F), 7–23 y	LCR	CVM:^9^ 6 CS	DHEAS	Saliva	DHEAS levels increased progressively from CS1 to CS2, sharp increase at CS3, followed by a gradual increase to reach peak at CS6 Strong positive correlation coefficient of 0.94 btw CS and DHEAS levels
4.	Sookhakian et al., 2022^40^	CSS	55, 7–20 y	LCR	CVM:^9^ 6 CS	IGF-1, ALP	Saliva	Strong positive correlation btw chronological age and CVM staging (*r*=0:836, *P*<0.001) Regression model with IGF-1, ALP, and chronological age provided best skeletal growth prediction (*P*<0.001) Predictive potential of model for pre-puberty (95%), puberty (80%), post-puberty (90%)
5.	Tsagkari, 2022^41^	CSS	54	LCR	CVM:^9^ 3 groups: pre-puberty (CS1, CS2), puberty (CS3, CS4), post-puberty (CS5, CS6)	Metabolomics	Saliva	61 endogenous compounds identified Significant difference for glycerol (*P*<0.01), glyceric acid (*P*<0.05), btw pre-puberty and puberty; mannose differed (*P*=0.12) btw pre-puberty and post-puberty, glucose differed btw puberty and post-puberty, and glutamic acid btw pre-puberty and puberty groups, puberty and post-puberty groups Metabolite differences with dental and chronological age also identified
6.	Carelli et al., 2021^42^	CSS	37 (17 M, 20 F), 10-16.3 y, class II patients with retrognathic mandible	CBCT, hand-wrist	CVM:^31^ 6 CS on LCR and 11 SMI^6^ on hand-wrist: stage 1, SMI 1–3 (pre-puberty); stage 2, SMI 4–5 (toward peak puberty); stage 3, SMI 6–8 (peak puberty); stage 4, SMI 9–10 (decelerating growth), stage 4, SMI 11 (post-puberty)	IGF-1	Serum	Strong correlation btw CVM staging and hand-wrist staging, but moderate correlation btw IGF-1 levels and CVM/SMI staging
7.	Kahlon et al., 2021^43^	CSS	240 (120 M, 120 F), 8-16 y, 20 subjects in each grp	LCR	CVM:^11^ 3 groups: pre-puberty (CS1, CS2); puberty (CS3, CS4); post-puberty (CS5, CS6)	IGF-1	Serum	IGF-1 levels increase from CVM stage 1 to reach peak at CVM 3 and fall later till CVM 6 in both M and F
8.	Wen et al., 2021^24^	CSS	66 (35 M, 31 F) (32 class I, 11.8±2.4 y), (34 class II, 12.2±2.4 y)	LCR	CVM:^9^ 3 grp: pre-puberty (CS1, CS2); puberty (CS3, CS4); post-puberty (CS5, CS6)	TF, DBP	GCF	Percentage TF, DBP in GCF is significantly higher in puberty than pre-pubertal or post-pubertal stage both in maxilla and mandible
9.	Anusuya et al., 2020^44^	CSS	90 (45 M, 45 F), 7-21 y	LCR	CVM:^9^ 6 CS, pre-puberty (CS1, CS2); puberty (CS3, CS4); post-puberty (CS5, CS6)	DHEAS, IGF-1	Serum	DHEAS, IGF-1 levels reach peak in males at CS4 and in females at CS3 (IGF-1, DHEAS levels in puberty significantly higher than pre-puberty stages). Serum DHEAS in males significantly higher than in females for all CS except CS2 and CS3. Serum IGF-1 in males significantly higher than females in all CS except CS1 and CS3
10.	Alhazmi et al., 2019^27^	CSS	79 (31 M, 48 F), 7-23 y	LCR	5 CS:^10^ CS 1,2,3,4,5	ALP activity, protein conc.	Saliva	Salivary ALP higher in CS1 Salivary ALP significantly different btw CS1-2 and CS1-5, peak at early puberty stage IQR protein conc. peak at CS3 and CS5 Positive significant correlation btw chronological age and CS
11.	Tripathi et al., 2019^20^	Longitud.	63, 11–17 y	LCR	6 CVMI stages^12^ (CVMI 1-6)	BALP, IGF-1	Serum	Peak IGF-1, BALP levels at CVMI 3, BALP reaches peak earlier than IGF-1 Positive correlation btw IGF-1 and BALP, but BALP more accurate for skeletal maturity IGF-1 and BALP annual % changes in negative correlation with mandibular length
12.	Wijaya et al., 2019^23^	CSS	136 (64 M, 72 F), 8-18 y	LCR	CVM.^9^ 3 grps: pre-puberty/pre-peak puberty (CS1, CS2), puberty/peak puberty (CS3, CS4), post-puberty/post-peak puberty (CS5, CS6)	BALP and total protein	Saliva	Pubertal phase prediction using multinomial logistic regression including chronological age, salivary BALP levels, and body mass index percentile Salivary BALP level in pre-peak puberty grp is greater than in peak puberty grp, which in turn is greater than in post-puberty grp
13.	Wen et al., 2018^45^	CSS	40 (20 M, 20 F); puberty grp (10 M, 10 F) mean age (9.2±1.4 y); post-puberty grp (10 M, 10 F) mean age (23.3±0.64 y)	LCR	CVM:^10^ puberty (CS3 and 4), post-puberty (CS5 and 6)	Protein conc., DBP, TF	GCF	Of 537 total identified proteins in GCF, 25 were upregulated and 18 downregulated in puberty versus post-puberty grp DBP and TF in puberty grp are significantly higher in than post-puberty grp
14.	Tripathi et al., 2018^21^	CSS	150 (75 M, 75 F), 8–20 y	LCR	6 CVMI stages^12^ (CVMI 1–6)	BALP and IGF-1	Serum	Peak serum IGF-1 levels: M=CVMI 4; F=CVMI 3 Peak serum BALP: CVMI 3 for both sexes Statistically significant correlation btw serum IGF-1 and serum BALP across CVMI 1–3 and 4–6 (*P*<0.01)
15.	Tripathi et al., 2017^22^	CSS	150 (75 M, 75 F), 8–20 y	LCR	6 CVMI stages^12^ (CVMI 1–6)	OC, IGF-1	Serum	Peak IGF-1 at CVMI 4 and 3, peak OC at CVMI 5 and 3 in M and F, respectively Statistically significant correlation btw IGF-1 and OC in CVMI 1–6 in M (*P*<0.05), and CVMI 3-6, 4–6 in F (*P*<0.05)
16.	Jain et al., 2017^46^	CSS	90 F, 8–20 y	LCR	6 CVMI stages^12^ (CVMI 1–6)	IGF-1, IGFBP-3, and its ratio	Serum	Serum IGF-1 increase across CVMI 1-3, peak levels (403.3±12.3 ng/mL), then decrease across CVMI 3-6; serum IGFBP-3 increase across CVMI 1-4 (peak values 5186.8±1384.2 ng/mL), then decrease across CVMI 4-6 High positive correlation btw IGF-1 and IGFBP-3 to CVMI 1–3
17.	Venkatagiriappa et al., 2016^47^	CSS	107 (45 M, 62 F) 5–25 y	Hand-wrist	11 SMI:^6^ Stage 1, SMI 1–3 (pre-puberty); stage 2, SMI 4–5 (toward peak puberty); stage 3, SMI 6–8 (peak puberty); stage 4, SMI 9–10 (decelerating growth); stage 4, SMI 11 (post-puberty)	DHEAS	Blood	Mean DHEAS levels increase from pre-puberty (75.95 μg/dL), to post-puberty (102.24 μg/dL), but not statistically significant at *P*<0.05
18.	Giuseppe et al., 2016^25^	CSS	100 (38 M, 62 F), mean age, 11.5±2.4 y; range, 7.6–17.7 y)	LCR	CVM:^9^ 3 grps: Grp A, pre-puberty (CS1, CS2); Grp B, puberty (CS3, CS4); Grp C, post-puberty (CS5, CS6)	ALP	GCF	GCF ALP levels in puberty grp two times greater than in pre-puberty/post-puberty grp (*P*<0.001) Intermediate mixed dentition only noted in pre-puberty grp (*P*<0.001) Negative correlation of GCF ALP levels with pre-puberty and post-puberty grp
19.	Sinha et al., 2016^48^	CSS	72 F, 8–20 y	LCR	6 CVMI stages^12^ (CVMI 1–6)	IGF-1	Serum and urine	Median serum IGF-1 levels increased across CVMI 1–4 (statistically significant peak at 408.59 ng/mL), decreased later Urine IGF-1 levels also showed peak at CVMI 4 (5.12 ng/mL)
20.	Gupta et al., 2015^49^	CSS	60 (30 M, 30 F), 8–23 y	LCR	CVM:^9^ 6 CS (CS1–6)	IGF-1	Serum	M: serum IGF-1 increased across CS1–4, then decreased F: increased across CS1–3, then decreased Greatest mean IGF-1 value in F (397 ng/mL) greater than in M (394.8 ng/mL)
21.	Masoud et al., 2015^50^	Longitud.	25 (13 M, 12 F), 9.2–17.4 y, 43 yearly samples	LCR	CVM:^9^ 3 grps: Grp A, pre-puberty (CS1, CS2); Grp B, puberty (CS3, CS4); Grp C, post-puberty (CS5, CS6); single observer	IGF-1	Blood	Average IGF-1 level and IGF-1 change were significantly associated with increase in mandibular length across the grps
22.	Masoud et al., 2015^51^	Longitud.	25, (13 M, 12 F) 9.2–17.4 y; 43 yearly samples	LCR	CVM:^9^ 3 grps: Grp A, pre-puberty (CS1, CS2); Grp B, puberty (CS3, CS4); Grp C, post-puberty (CS5, CS6); single observer	IGF-1	Blood	Moderate positive correlation btw total AFH change and annual % change in blood spot IGF-1 levels (*P*<0.01)
23.	Nayak et al., 2014^52^	CSS	45 (21 M, 24 F), 7–23 y	LCR	QCVM staging:^32^ QCVM I/accelerating velocity (*n*=13); QCVM II/high velocity (*n*=15); QCVM III/decelerating velocity (*n*=10); QCVM IV/completing velocity (*n*=7)	IGF-1 and salivary secretion rate	Saliva	IGF-1 levels in QCVM II greater than QCVM I (*P*<0.0001), and QCVM II greater than QCVM III/IV (*P*<0.001), at completing velocity stage
24.	Jain et al., 2013^53^	CSS	45 M	LCR	CVM:^9^ CS3, 4, 5 (*n*=15 for each CS) divided into 3 grps: advanced, average, and delayed maturers	IGF-1	Serum	Peak IGF-1 level in CS4, in CS5 IGF-1 level low in all 3 maturity grps
25.	Hussain et al., 2013^54^	CSS	90 (47 M, 43 F), 5–20 y	LCR	CVM:^9^ CS1–6 (*n*=15 for each CS)	PTHrP	Serum	Serum PTHrP increased across CS1-5, peaks at CS5, declines sharply at CS6
26.	Gupta et al., 2012^55^	CSS	30 F, 8–23 y	LCR and IOPA	CVM:^9^ CS1–6, MP3^33^ (MP3F, MP3FG, MP3G, MP3H, MP3HI, MP3I)	IGF-1	Serum	Mean IGF-1 in CS1 (216±7.53 ng/mL) increased to peak levels in CS3 (397±20.76 ng/mL), then declined Mean IGF-1 in MP3F stage (218±8.73 ng/mL), increased to peak in MP3G stage (397±20.76 ng/mL), then declined
27.	Ishaq et al., 2012^56^	CSS	120 (60 M, 60 F); M, 10–18 y; F, 8–16 y	LCR	6 CS^12^ (CS1–6)	IGF-1	Serum	M: peak mean IGF-1 value in CS4 (mean age 14.5 y, 893±171 ng/mL) F: peak mean IGF-1 value in CS4 (mean age 14 y, 794±217 ng/mL) Sex-related differences at CS3 and 5
28.	Masoud et al., 2012^57^	Longitud.	25 (13 M, 12 F); 9.2–17.4 y; 43 yearly samples	LCR	CVM:^9^ 3 grps: Grp A: pre-puberty (CS1, CS2), Grp B: puberty (CS3, CS4), Grp C: post-puberty (CS5, CS6)	IGF-1	Blood	Significant mild to moderate correlation btw change in mandibular length and mean IGF-1 change in levels (*r*=0.4; *P*=0.008) Grp with ascending IGF-1 levels had moderate to high correlation with change in mandibular length (*r*=0.655; *P*=0.015)
29.	Srinivasan et al., 2012^58^	CSS	60 (30 F, 30 M); 7–30 y	Hand-wrist	Grp A: pre-puberty, Grp B: puberty, Grp C: post-puberty^34^,^35^ (*n*=20, 10 M, 10 F in each grp)	DHEAS	Serum	Mean DHEAS values in pre-puberty grp (0.43 μg/mL) is less than puberty grp (2.17 μg/mL) which in turn is lower than post-puberty grp (4.60 μg/mL) No significant difference btw M and F
30.	Perinetti et al., 2012^59^	CSS	50 (19 M, 31 F), 7.8-17.7 y	LCR	CVM:^9^ 6 CS; 3 grps: pre-puberty (CS1, CS2), puberty (CS3, CS4), post-puberty (CS5, CS6)	ALP activity, protein conc.	GCF	Total GCF ALP activity greatest in puberty grp compared to pre- and post-puberty grps Total protein and normalized GCF ALP activity showed no association with stages
31.	Perinetti et al., 2011^60^	CSS	72 (27 M, 45 F); 7.8–17.7 y	LCR	CVM:^9^ 6 CS; 3 grps: pre-puberty (CS1, CS2), puberty (CS3, CS4), post-puberty (CS5, CS6)	ALP activity	GCF	Two-fold peak of GCF ALP activity in puberty compared to pre- and post-puberty grps Sites: ALP activity in CS4 in maxillary sites and CS3 in mandibular sites is significantly greater than in CS2 stage
32.	Masoud et al., 2009^61^	CSS	84 (45 F, 39 M); 5–25 y	Hand-wrist	11 SMI:^6^ stage 1, SMI 1–3 (pre-puberty); stage, SMI 4–5 (toward peak puberty); stage 3, SMI 6–8 (peak puberty); stage 4, SMI 9–10 (decelerating growth); stage 5, SMI 11 (post-puberty)	IGF-1	Blood	IGF-1 in high velocity/peak puberty stage greater than accelerating (stage 2)/pre-puberty (stage 1)/post-puberty (stage 5)
33.	Masoud et al., 2008^62^	CSS	83 (39 M, 44 F); 5–25 y	LCR	CVM:^9^ 6 CS; 3 grps: pre-puberty (CS1, CS2), puberty (CS3, CS4), post-puberty (CS5, CS6)	IGF-1	Blood	Mean blood spot IGF-1 levels in CS4 is significantly higher than in CS1, CS2, CS6 IGF-l levels show positive correlation upon progress from pre-pubertal to pubertal stages followed by negative correlation on attaining post-pubertal stage Negative correlation of IGF-1 levels with time of puberty onset and chronological age

AFH, anterior facial height; ALP, alkaline phosphatase; BALP, bone alkaline phosphatase; btw, between; CBCT, cone beam computed tomography; conc., concentration; Class I, normal upper and lower jaw relation; Class II, mandible retrognathic or maxilla prognathic or combination of both; CS, cervical stage(s); CSS, cross-sectional study; CVM, cervical vertebral maturation; CVMI, cervical vertebral maturation index; CVS, cervical vertebral staging; DBP, vitamin D binding protein; DHEAS, dehydroepiandrosterone sulfate; F, Female; grp(s), group(s); GCF, gingival crevicular fluid; IGF-1, insulin growth factor-1; IGFBP-3, insulin-like growth factor binding-protein-3; IOPA, intra-oral periapical radiograph; IQR, interquartile range; LCR, lateral cephalometric radiograph; Longitud., longitudinal; M, male;); MP3F, middle phalanx of third finger (F stage); MP3FG, middle phalanx of third finger (FG stage); MP3G, middle phalanx of third finger (G stage); MP3H, middle phalanx of third finger (H stage); MP3HI, middle phalanx of third finger (HI stage); MP3I,middle phalanx of third finger (I stage); OC, osteocalcin; PTHrP, parathormone-related protein; QCVM, quantitative cervical vertebral maturation; SMI, skeletal maturity indicators; TF, serotransferrin; y, year(s); X-ray, radiograph.

**Table 2:** Biofluid Collection and Biomarker Analysis.

#	Study	Biofluid	Oral Hygiene Protocol	Gingival and Periodontal Inflammation	Biofluid Collection		Analysis Method
					Time	Protocol	
1.	Almaki, 2022^37^	Saliva	NM	OHI-ULS, BOP, GBI, PD, CAL, CPI	10.00–12.00	5 mL unstimulated saliva, spitting method, stored at −20°C, centrifuge	ELISA
2.	Almaki et al., 2022^38^	Saliva	Yes	OHI-S, BOP, PD, CAL, CPI	9.00–10.00	5 mL unstimulated saliva, spitting method, stored at −80°C, centrifuge	ELISA
3.	Al Meshari et al., 2022^39^	Saliva	Yes	PD, plaque and gingival indices	8.00–11.00	1–5 mL unstimulated saliva, passive drool, stored at −80°C, centrifuge	ELISA
4.	Sookhakian et al., 2022^40^	Saliva	NM	NM	8.00–11.00	Unstimulated saliva, spitting method,	ELISA
5.	Tsagkari, 2022^41^	Saliva	Yes, PD exam, OHIn, ULS 2 wk before saliva coll.	Plaque index, bleeding index	NM	1.5 mL unstimulated saliva, stored at −80°C, centrifuge	GC-MS
6.	Carelli et al., 2021^42^	Serum	NM	NM	NM	5 mL blood collected week of X-ray, centrifuge for serum separation	ELISA
7.	Kahlon et al., 2021^43^	Serum	NM	NM	9.00–12.00	Blood collected from median cubital vein, centrifuged for serum	ELISA
8.	Wen et al., 2021^24^	GCF	Yes+OHIn 1 wk before GCF coll.	PD, BI, AL measurement	8.00–10.00	ML and DL sites of maxillary and mandibular central incisors, PP, insertion for 60 s, repeat after 2 min, stored at −80°C	SP for total protein, ELISA for TF and DBP
9.	Anusuya et al., 2020^44^	Serum	NM	NM	9.00–12.00	3 mL blood drawn from median cubital vein	ELISA
10.	Alhazmi et al., 2019^27^	Saliva	NM	NM	9.00–12.00	Unstimulated whole saliva, passive drool, 1–5 mL, stored at −80°C	ELISA
11.	Tripathi et al., 2019^20^	Serum	NM	NM	9.00–10.00	5 mL venous blood collected, pipetted equally for IGF-1 and BALP, stored at −80°C	ELISA
12.	Wijaya et al., 2019^23^	Saliva	Yes with OP (supra-G, sub-G scaling) 1 wk before saliva coll.+OHIn	NM	Morning sample	Passive drool, ice-box transfer, centrifuge	Bradford Assay Kit and ELISA
13.	Wen et al., 2018^45^	GCF	Yes with OHIn 1 wk before GCF coll.	PD, BI, AL measurement	8.00–10.00	Paper points, left for 30 s, 4 PP at one site, centrifuge at 4°C	SDS-PAGE, tandem mass tag labeling coupled with LC-MS, ELISA for DBP and TF
14.	Tripathi et al., 2018^21^	Serum	NM	NM	NM	5 mL venous blood collected, pipetted, stored at −80°C	ELISA
15.	Tripathi et al., 2017^22^	Serum	NM	NM	9.00–10.00	5 mL venous blood collected, pipetted, stored at −80°C	ELISA
16.	Jain et al., 2017^46^	Serum	NM	NM	9.00–11.00	2.5 mL venous blood collected, pipetted, stored at −70°C	ELISA
17.	Venkatagiriappa et al., 2016^47^	Blood	NM	NM	NM	Dried bloodspot kit, stored at −70°C	Immuno-assay
18.	Giuseppe et al., 2016^25^	GCF	Yes with OHIn 1 wk before GCF coll.	PD, BI, AL measurement	NM	Mesial and distal sites of mandibular central incisors, PP, insertion 1 mm for 60 s, stored at −80°C	SP
19.	Sinha et al., 2016^48^	Serum and urine	NM	NM	NM	5 mL venous blood collected, pipetted for IGF-1, stored at −80°C	ELISA
20.	Gupta et al., 2015^49^	Serum	NM	NM	12.00–15.00	Blood drawn from median cubital vein, stored btw 2–8°C	CLA
21.	Masoud et al., 2015^50^	Blood	NM	NM	NM	Dried blood spot kit, stored at −18°C	RIA
22.	Masoud et al., 2015^51^	Blood	NM	NM	NM	Dried blood spot kit, stored at −18°C	RIA
23.	Nayak et al., 2014^52^	Saliva	NM	NM	NM	Parotic saliva, Lashley cup, stored at −20°C	IRMA
24.	Jain et al., 2013^53^	Serum	NM	NM	12.00–15.00	Blood drawn, stored btw 2–8°C	Two-site CLA
25.	Hussain et al., 2013^54^	Serum	NM	NM	NM	3 mL blood drawn from median cubital vein, stored at −20°C	ELISA
26.	Gupta et al., 2012^55^	Serum	NM	NM	NM	Blood drawn, stored btw 2–8°C	Two-site CLA
27.	Ishaq et al., 2012^56^	Serum	NM	NM	9.00–11.00	Blood drawn, stored at −20°C	ELISA
28.	Masoud et al., 2012^57^	Blood	NM	NM	NM	Dried blood spot kit, stored at −18°C	RIA
29.	Srinivasan et al., 2012^58^	Serum	NM	NM	NM	2.5 mL of venous blood drawn, stored at −20°C	ELISA
30.	Perinetti et al., 2012^59^	GCF	Yes with OHIn 1 wk before GCF coll.	PD, BI, AL measurement	8.00–10.00	Mesial and distal sites of mandibular and maxillary central incisors, PP, insertion 1 mm for 60 s, stored at −80°C	SP
31.	Perinetti et al., 2011^60^	GCF	Yes with OHIn 1 wk before GCF coll.	PD, BI, AL measurement	8.00–10.00	Mesial and distal sites of mandibular and maxillary central incisors, PP, insertion 1 mm for 60 s, stored at −80°C	SP
32.	Masoud et al., 2009^61^	Blood	NM	NM	NM	Dried blood spot kit, stored at −20°C	RIA
33.	Masoud et al., 2008^62^	Blood	NM	NM	NM	Dried blood spot kit, stored at −20°C	RIA

AL, attachment loss; BI, bleeding index; BOP, bleeding on probing; btw, between; CAL, clinical attachment loss; CLA, chemiluminescent assay; coll., collection; CPI, community periodontal index; DBP, vitamin D binding protein; DL, distolabial; ELISA, enzyme-linked immunosorbent assay; GBI, gingival bleeding index; GCF, gingival crevicular fluid; GC-MS, gas chromatography-mass spectrometry; IRMA, immunoradiometric assay; LC-MS, liquid chromatography-mass spectrometry; min, minute(s); ML, mesiolabial; NM, not mentioned; OH, oral hygiene; OHI, oral hygiene index; OHIn, oral hygiene instruction; OHI-S, simplified oral hygiene index; OP, oral prophylaxis; PD, periodontal depth; PP, paper points; RIA, radioimmunoassay; s, second(s); SDS-PAGE, sodium dodecyl sulphate-polyacrylamide gel electrophoresis; SP, spectrophotometer; sub-G, sub-gingival; supra-G, supra-gingival; TF, serotransferrin; ULS, ultrasonic scaling; wk, week(s); X-ray, radiograph.

### Risk of Bias/Quality Assessment

The risk of bias (ROB) and applicability testing of all the studies (*n*=41) was done by two observers independently (PK and RB) with the modified QUADAS-2 tool,^30^ using the four primary domains (Supplementary Figure 1). Domain 2 (index test) was performed separately for each body fluid: saliva, GCF, blood/serum, and urine. The reference standard for skeletal maturity assessment was radiographic indices using lateral cephalogram (CS/CVM staging),^9^,^10^,^12^,^31^,^32^ intraoral periapical radiograph (IOPA) (middle phalanx of the third finger [MP3]),^7^,^11^,^33^ and hand-wrist (skeletal maturity indicators [SMI]).^6^,^34^–^36^

**Figure 1:**
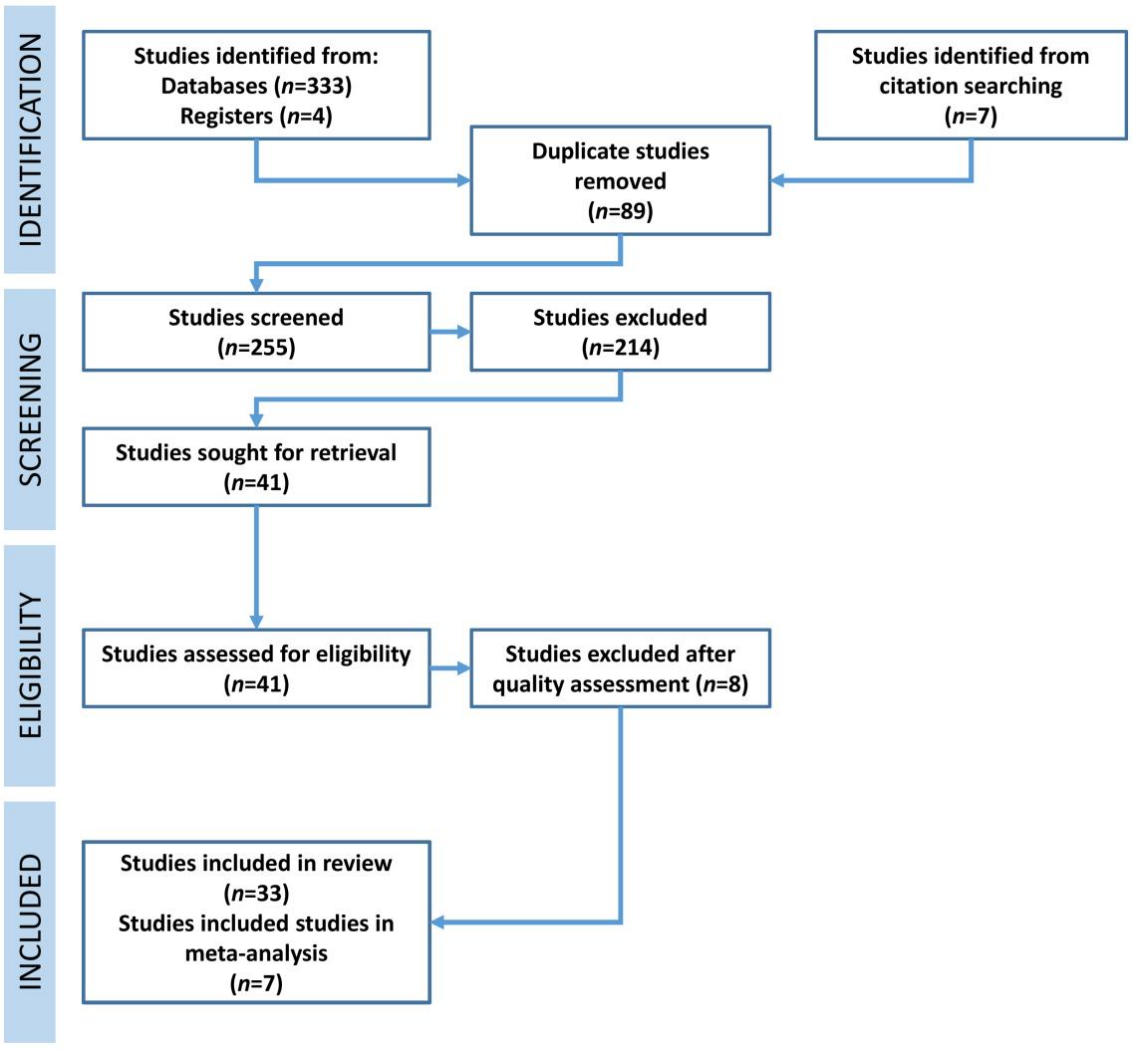
PRISMA Flowchart Outlining the Selection of Studies for this Review and the Meta-analysis.

The quality assessment by QUADAS-2 found 33 out of a total of 41 studies to be eligible for inclusion in the review.^20^–^25^,^27^,^37^–^62^ The eight studies that were excluded were based on an unclear ROB,^63^ high ROB,^64^ and an unclear ROB and applicability.^65^–^70^

The final inclusion of articles in the review after quality assessment are presented in a PRISMA flow diagram (Figure 1).^71^

#### Data extraction

Two observers (PK and RB) performed the detailed data extraction independently (Tables 1 and 2). Any discordance in the findings was discussed with two other investigators (OPK and AC), and a consensus was reached.

#### Meta-analysis

Due to heterogeneity in studies, meta-analysis was not possible for all the studies but only for studies evaluating IGF-1 levels in serum among CVMI 2, 3, 4, and 5. Seven studies 22,43,44,46,48,53,56 qualified for the meta-analysis based on evaluation method (ELISA), unit of measurement (ng/mL), and availability of mean, SD (standard deviation), and sample size. The meta-analysis was performed separately for males and females, with five studies^22^,^43^,^44^,^53^,^56^ and six studies^22^,^43^,^44^,^46^,^48^,^56^ included for evaluation of IGF-1, respectively. In the studies for males, one study^53^ was considered only for assessment of CVMI 3, 4, and 5 based on the availability of data.

#### Statistical analysis

The meta-analysis was performed using RevMan (version 5.4) software. The heterogeneity and chi-square tests were performed, and a 95% confidence interval was calculated and graphically represented. A random-effect model was utilized to reduce the existing variability, and forest plots were generated to graphically represent the weighted mean difference and overall test for significance.

## RESULTS (TABLE 1)

### Participants

The number of subjects evaluated in the different studies were in the ranges 1–50 (9 studies), 51–100 (15 studies), and 101 and above (9 studies). The male-to-female ratio was unequal in 17 studies, with only female subjects recruited in three studies^46^,^48^,^55^ and only male subjects in one study.^53^

### Study Characteristics (Table 1)

Studies employed different indices for SME; the CVM staging developed by Baccetti et al.^9^ was used in 18 studies (Table 1). 23–25,37,39–41,44,49–51,53–55,57,59,60,62 This was followed by using the six cervical stages of Hassel and Farman^12^ in six studies;^20^–^22^,^46^,^48^,^56^ of Baccetti et al.^10^ in two studies;^27^,^45^ of Hagg and Taranger^11^ in one study;^43^ of McNamara^31^ in one study;^42^ and using the quantitative cervical vertebral maturation (QCVM) method by Chen.^52^ Hand-wrist radiographs used the Fishman index^6^ with 11 SMIs^42^,^47^,^61^ and five SMI stages.^38^ The study design was cross-sectional in most studies and longitudinal in four studies with a repeated collection of biomarkers.^20^,^50^,^51^,^57^

### Collection of Body Fluids and Handling (Tables 1 and 2)

Different body fluids were used, including blood and serum in 20 studies, GCF in four studies, saliva in eight studies, and urine in one study. Studies specified the time of sample collection in 17 out of 34 studies: between 09.00 and 12.00, or between 12.00 and 15.00. Saliva and GCF collection studies involved oral prophylaxis, regimen instructions, and gingival and periodontal indices for oral hygiene assessment. The sample collection of GCF was done by paper points, saliva by passive drool or parotid saliva, and blood by venous blood or dried blood spot collection. Biomarker analysis was done by enzyme-linked immunosorbent assay (ELISA), radioimmunoassay, spectrophotometry, two site chemiluminescent assay, liquid chromatography–mass spectrometry, immunoassay, gas chromatography–mass spectrometry, and immunoradiometric assay.

### Levels of Biomarkers in Association with Skeletal Maturation (Tables 1 and 2)

The biomarkers investigated in most of the studies were IGF-1,^20^–^22^, ^37^, ^38^, ^40^, ^42^–^44^, ^46^, ^49^–^51^, ^53^, ^55^–^57^, ^61^, ^62^ ALP,^25^,^27^,^40^,^59^,^60^ dehydroepiandrosterone sulfate (DHEAS),^39^,^44^,^47^,^58^ and BALP,^20^,^21^,^23^ with sporadic assessment of DBP^24^,^45^ and TF,^24^,^45^ IGF binding protein (IGFBP-3),^37^,^38^,^46^ IGF-1/IGFBP-3 ratio,^37^,^38^,^46^ osteocalcin,^22^ PTHrP,^54^ and metabolites.^41^

#### Insulin-like growth factor-1

In serum and saliva, IGF-1 showed a statistically significant rise in the pubertal (CS3, CS4), compared with pre-pubertal (CS1, CS2) and post-pubertal stages (CS5, CS6).^7^,^43^,^27^,^21^,^20^,^22^,^53^,^46^,^48^,^55^,^56^,^62^

The peak levels corresponded with high-velocity stages (SMIs 6–8) on hand-wrist radiographs^38^,^61^ and at MP3 stage on IOPA.^55^

The sex-related difference in IGF-1 showed an early onset of puberty in females with peak IGF-1 at CVMI 3, while males showed a peak at CVMI 4 followed by a sustained rise in levels.^21^,^22^,^37^ Peak levels varied in both sexes, with levels higher in females^49^ and in males^22^ in one study each.

A positive correlation of IGF-1 was established with varied biomarkers across different skeletal stages. A correlation was documented with BALP,^20^ osteocalcin across CVMI 1–4 (males)^22^ and CS1–3 (females),^22^ and IGFBP-3 across CVMI 1–3.^46^ Also, IGF-1 levels in serum and urine levels showed a positive correlation in CS 1–4.^48^ In saliva, QCVM II (high velocity) shows higher IGF-1 levels than QCVM I, III, or IV.^52^ With the chronological age, IGF-1 showed a positive correlation till CS3, followed by a negative correlation at CS4 and CS5.^53^

Many longitudinal studies found a positive association of IGF-1 with maxillo-mandibular growth parameters, anterior facial height, mandibular plane, and the type of orthodontic forces.^20^,^51^,^52^,^57^

#### Bone-specific alkaline phosphatase

Levels of BALP peaked at CVMI 3 in both sexes, but slightly earlier than IGF-1.^20^,^21^ Serum IGF-1 and BALP showed a statistically significant correlation (*P*<0.01),^20^,^21^ but the latter was considered more accurate for skeletal maturation.^20^

#### Osteocalcin

The mean serum osteocalcin levels showed distinction in sex, with an increase from CVMI 1 to CVMI 5 in males and from CVMI 1 to CVMI 3 in females, following the levels of IGF-1 across the skeletal stages but showing no significant variation across stages. The levels were higher in males than in females. A significant correlation between IGF-1 and osteocalcin was seen in different skeletal stages, both in males and in females (*P*<0.05).^22^

#### Vitamin-D binding protein and serotransferrin

Studies in GCF showed a significantly higher percentage of DBP and TF in pubertal as compared to pre- or post-pubertal stage in both maxillary and mandibular incisors.^24^,^45^ But no difference in TF and DBP levels was found with malocclusion (class I and class II) or sex.^24^

#### Dehydroepiandrosterone sulfate

The levels of DHEAS were higher in pubertal compared to pre-pubertal stages in one study,^44^ while other studies showed a non-significant increase in mean DHEAS levels from pre-pubertal to post-pubertal stage.^39^,^47^,^58^ Additionally, males showed a higher peak serum DHEAS at CS4 (685.33±39.11 nmol/mL) than the female peak at CS3 (578.12± 13.76 nmol/mL).^44^

#### Alkaline phosphatase

Evaluation of levels of ALP in saliva combined with chronological age was able to predict pubertal growth better as compared to evaluating the levels of salivary ALP alone.^27^ The level of salivary ALP activity in CS2 (*P*<0.001) and CS5 (*P*=0.004) was significantly higher than at stage 1. In contrast, the total ALP protein concentration in saliva was highest at CS3 and CS5 as compared to other stages.^27^ Level of ALP was lower in females than in males.^27^ Levels of ALP and activity in GCF were twice as high in the pubertal than in the pre-pubertal/post-pubertal stage, and a negative correlation of GCF ALP levels was established with the pre- and post-pubertal phase.^25^,^59^,^60^

#### Parathormone-related protein

Serum parathormone-related protein (PTHrP) levels followed a consistent pattern of increase from CS1 to CS5 with a correlation coefficient of 10.68 (*P*<0.001), a peak shown at CS5, and thereafter a sharp decline at CS6 (coefficient of 0.676). The correlation with age was significant at CS1 (*P*=0.03) and CS2 (*P*=0.005).^54^

#### Metabolomics

Metabolites like glycerol (*P*<0.01) and glyceric acid (*P*<0.05) showed significant difference between pre-pubertal and post-pubertal stages. Pre-pubertal and post-pubertal stages showed difference in mannose (*P*=0.12) and pyroglutamic acid, while pubertal and post-pubertal stages showed difference in glucose and pyroglutamic acid.^41^ Besides, the metabolites also differed with dental and chronological age.

The associations of marker levels with skeletal staging, sex, craniofacial parameters, and their significant inter-relationships have been compiled in Table 3.

**Table 3:** Results Associated with Variation in Mediator Levels in Different Skeletal Maturation Stages.

Mediator	Criteria	Results
Insulin-like growth factor-1 (IGF-1)	Upregulation/downregulation	Level in pubertal stage (CS3, CS4) greater than in pre-pubertal (CS1, CS2)/pubertal (CS5, CS6) stages^20^–^22^,^37^,^43^,^46^,^48^,^53^,^56^,^62^ Levels in high velocity stages (SMI 6–8) greater than in accelerating stage (SMI 4–5)/pre-pubertal (SMI 1–3)/decelerating velocity (SMI 9–10)/post-pubertal stages (SMI 11)^38^,^61^ Salivary IGF-1 at QCVM II (high velocity) greater than in QCVM I (accelerating velocity)/QCVM III (decelerating)/QCVM IV (completed)^52^
	Peak levels	CS3 stage^20^,^37^,^43^,^46^,^55^ CS4 stage^37^,^43^,^48^,^58^,^62^
	Sex predilection	Peak IGF-1 levels in F at CVMI 3;^20^–^22^,^44^,^46^,^49^,^55^ CVMI 4^48^ Peak IGF-1 levels in M at CVMI 4^20^,^22^,^49^,^53^,^56^ Peak IGF-1 levels in M and F at CVMI 4^56^ Peak levels in F greater than in M,^49^ and in M greater than in F^23^,^37^,^38^
	Significant correlations	Positive correlation btw BALP and IGF-1 across various skeletal stages^20^ Positive correlation btw OC and IGF-1 in CVMI 1–6 (M) and CVMI 3–6,4–6 (F)^22^ Positive correlation btw IGF-1 and IGFBP-3 across CVMI 1–3^37^,^38^,^46^ Positive correlation of IGF-1 levels in serum and urine across CVMI 1–4^48^ Positive correlation of IGF-1 with age till CS3 and negative correlation until CS4, CS5^53^ Negative correlation of IGF-1 with time of pubertal onset and age^62^ IGF-1 peak levels at CS3 in correlation with peak IGF-1 levels at MP3G stage^45^
	Craniofacial parameters	Negative correlation of annual change in IGF-1% with mandibular length^20^ Positive correlation of IGF-1 increase with increasing mandibular length^50^ Positive correlation of AFH and mandibular plane change with % change in IGF-1 levels^50^ Moderate to high correlation of ascending IGF-1 levels (> 250 μg/L) to greater mandibular growth (5.6 mm)^57^
	Growth predictive model	Regression model with IGF-1, ALP, and chronological age provided the best skeletal growth prediction (*P*<0.001)^40^
Bone-specific alkaline phosphatase (BALP)	Upregulation/downregulation	BALP levels peak at CVMI 3 in both sexes^20^,^21^ Negative correlation of annual % change in BALP with mandibular length^20^ Salivary BALP levels in pre-pubertal stage greater in the pubertal stage greater than in post-pubertal stage^21^
	Association with other parameters	BALP levels peak at CVMI 3 earlier than IGF-1^20^,^21^ BALP more accurate than IGF-1 for skeletal maturation^20^
Osteocalcin (OC)	Upregulation/downregulation	Mean serum OC levels reach peak at CVMI 5 in M, and from CVMI 3^22^ OC levels in M greater than in F^22^
	Correlations	Significant correlation btw IGF-1 and OC in CVMI 1–6 (M) (*P*<0.05), and CVMI 3–6, 4–6 (F) (*P*<0.05)^22^
Vitamin D binding protein (DBP) and serotransferrin (TF)	Upregulation/downregulation	DBP and TF % in GCF in pubertal greater than in pubertal/post-pubertal stage^24^,^45^ In post-pubertal stage, mandibular TF greater than maxillary TF^24^ In pre-pubertal and post-pubertal stages, maxillary DBP greater than mandibular DBP^24^ No difference in TF and DBP levels in malocclusion class or sex^24^
	Pubertal predictive model	Maxillary TF levels and age important variables, maxillary TF more accurate btw pubertal and non-pubertal stages, with 100% specificity and 68.2% sensitivity^24^
Dehydroepiandrosterone sulfate (DHEAS)	Upregulation/downregulation	Levels of DHEAS in pubertal greater than in pre-pubertal^39^,^44^ Mean DHEAS levels steady increase from pre-pubertal to post-pubertal stage^39^,^47^,^58^
	Sex predilection	Peak in M at CS4, and in F at CS3^44^ Levels in M greater than in F at all stages except CS2, 3^44^
Alkaline phosphatase (ALP)	Upregulation/downregulation	Peak in early pubertal stage^27^ ALP protein concentration peaked at CVM stage 3 (1.44 [0.65] mg/mL) and CVM stage 5 (1.50 [0.46] mg/mL)^27^ GCF ALP levels in pubertal greater than in pre-pubertal/post-pubertal stage^25^,^60^ Total GCF ALP activity in pubertal greater than in non-pubertal stages^60^
	Correlations	Significant positive correlation btw salivary ALP and age^27^ Negative correlation of GCF ALP levels with pre-pubertal/post-pubertal phase^25^,^60^
	Pubertal predictive model	ALP and age combined proved better predictive accuracy for pubertal peak than ALP alone^27^
	Sex predilection	Levels in M greater than in F^27^
Parathormone-related protein (PTHrP)	Upregulation/downregulation	Serum PTHrP increase across CS1–5 with a correlation coefficient of 10.68 (*P*<0.001)^54^ Peak levels seen at CS5, then decline at CS6 (coefficient 0.676)^54^

AFH, anterior facial height; ALP, alkaline phosphatase; BALP, bone alkaline phosphatase; btw, between; CS, cervical stage; CVMI, cervical vertebral maturation index; DBP, vitamin D binding protein; F, female(s); GCF, gingival crevicular fluid; IGF-1, insulin-like growth factor; IGFBP-3, insulin-like growth factor binding protein-3; M, male(s); MP3G, middle phalanx of third finger (G stage); OC, osteocalcin; PTHrP, parathormone-related protein; QCVM, quantitative cervical vertebral maturation; SMI, skeletal maturity indicators; TF, serotransferrin.

### Outcome of Meta-analysis

**Figure 2:**
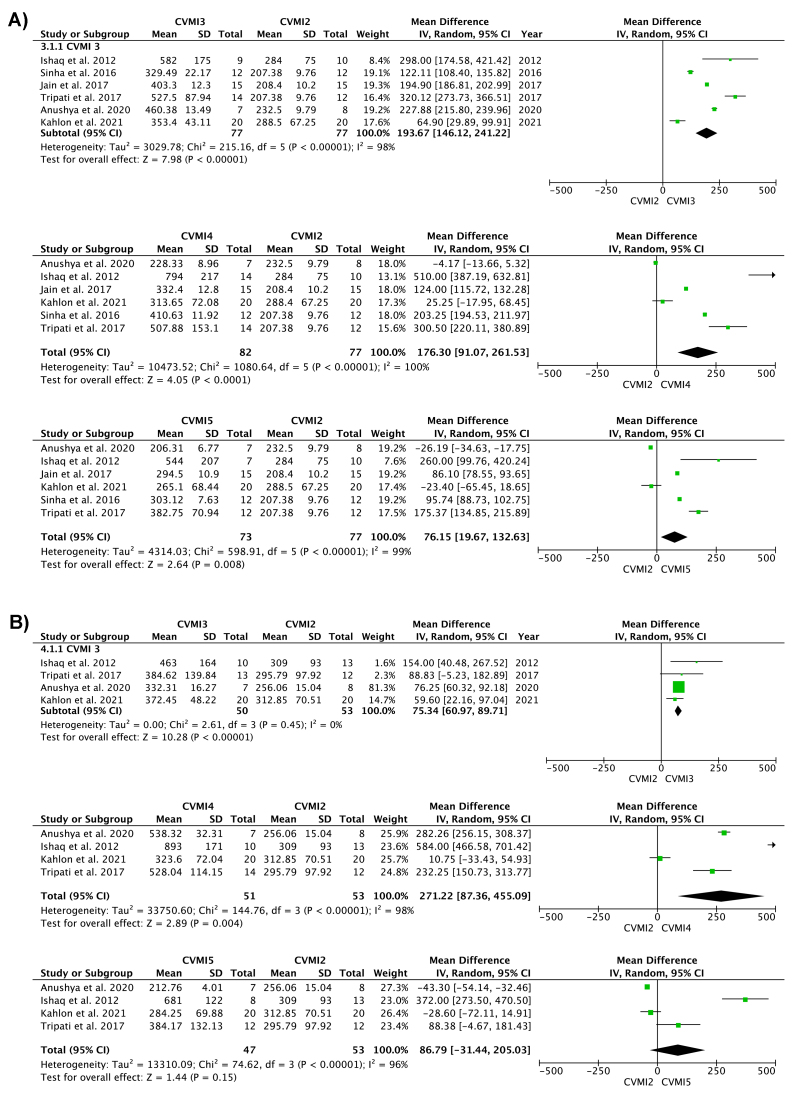
Forest Plots Showing (A) Results of 6 Studies in Females Showing Significant Rise of Serum IGF-1 from CVMI 2 to CVMI 3, 4, and 5, and (B) Results of 5 Studies in Males Showing Significant Rise of Serum IGF-1 from CVMI 2 to CVMI 3 and 4.

**Figure 3:**
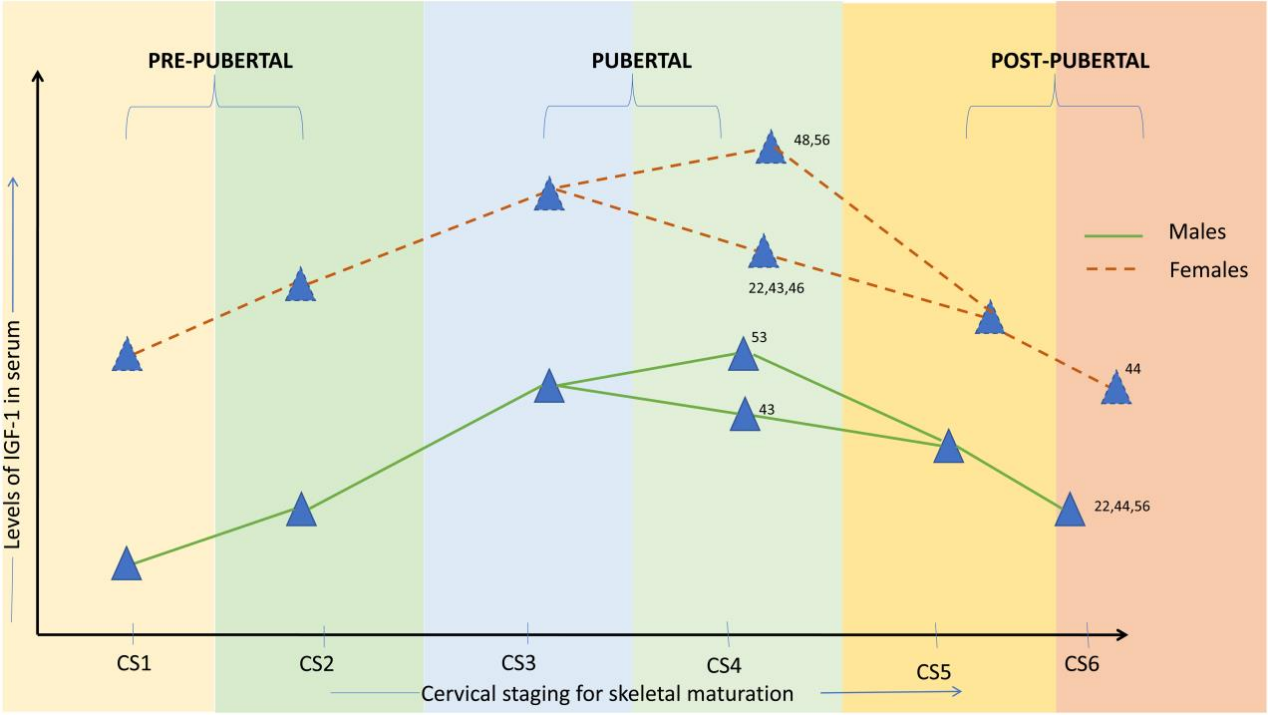
Pictorial Representation of Peak IGF-1 Levels in Both Males and Females for the Studies Included in the Meta-analysis (Tripathi,^22^ Kahlon,^43^ Anusuya,^44^ Jain,^46^ Sinha,^48^ Jain,^53^ and Ishaq^56^).

The random-effect model was used due to significant heterogeneity among the primary studies. The results of separate analysis for males and females depicted a highly significant rise in IGF-1 from CVMI 2 to CVMI 3, 4, and 5 in females (Figure 2A). In males, a highly significant rise in IGF-1 was seen from CVMI 2 to CVMI 3 and 4, but no significant difference was seen between CVMI 2 and 5 (Figure 2B). Supplementary Figure 2 (A and B) presents comparison of IGF-1 levels in males and females between CVMI 3, 4, and 5. A pictorial representation of peak IGF-1 levels in both males and females used in the meta-analysis is presented in Figure 3.

## DISCUSSION

The current meta-analysis explored the association of biomarkers in multiple body fluids (GCF, saliva, blood, serum, urine) with the stages of skeletal maturation as observed in CVM staging on lateral cephalograms or SMIs on hand-wrist radiographs. Due to the heterogeneity of data, we were able to perform meta-analysis for only seven studies evaluating IGF-1 in serum (Figure 2).^22^,^43^,^44^,^46^,^48^,^53^,^56^ Of these, five studies in only males and six studies in only females were included for analysis.

The meta-analysis in males showed significant rise of serum IGF-1 levels from CVMI 2 to CVMI 3 and 4, and in females from CVMI 2 to CVMI 3, 4, and 5. The CVMI 3 stage corresponds to the circum-pubertal stage of accelerating skeletal growth. The peak IGF-1 levels in CVMI 3 can be explained on the basis of blood IGF-1 having a role in influencing the replication of osteoprogenitor cells and their differentiation into mature osteoblasts by stimulating osteocalcin synthesis in bone.^72^,^73^ This can be confirmed by related literature evidence of a positive correlation of IGF-1 and osteocalcin, which is a marker for late osteoblastic differentiation in serum across all skeletal stages.^74^–^76^

A few studies on serum IGF-1 that did not qualify for meta-analysis have also shown peak serum IGF-1 at CS3,^46^ CS4,^62^ and at SMIs 6–8 (high growth velocity stage)^38^,^61^ on hand-wrist radiographs. This agrees with the results of the meta-analysis, showing a peak at CVMI 3 and no significant difference between CVMI 3 and 4, as all these stages correspond to heightened skeletal growth activity in growing individuals. Interestingly, the current review also indicates the serum growth hormone (GH)/IGF-1 ratio as a potent marker for skeletal maturation compared with IGF-1 alone. This is supported by literature evidence that IGF-1 is directly or indirectly influenced by GH production, and serum GH/IGF-1 ratio can assess growth and its deficiencies more accurately than IGF-1 levels alone.^77^,^78^ Besides, growth hormone is known to mediate maxillary and mandibular growth, which is designated by a positive correlation between IGF-1 level and its binding protein (IGFBP-3), as seen in one study included in the meta-analysis.^46^ A rise in IGFBP-3 implies increased biological activity of circulating IGF-1^79^ and is a more accurate marker of skeletal maturation compared to IGF-1, which may be explored in future SME studies.

A significant association has also been established between serum IGF-1 levels and anterior facial height in longitudinal evaluation in the current review.^50^ This can be explained by the influence of the strength of the masticatory apparatus and the evolution of dietary patterns on mid-face and mandibular growth, which is further based on genetics and environment.^80^ Ascending IGF-1 levels above 250 ng/mL were associated with greater mandibular growth compared to IGF-1 below 250 ng/mL.^49^,^50^ This can be of great clinical significance as timely assessments of IGF-1 levels can guide clinicians regarding the pattern of rise or fall of IGF-1, and related treatment alternatives to be selected.

The current review provides some interesting insights into IGF-1 cut-off levels and ratios that may require further exploration. Residual mandibular growth was depicted by higher IGF-1 levels after attaining CVMI 6 in one study,^57^ which can further assist orthodontists in planning orthopedic treatment in the late circum-pubertal stages of growth. The IGF-1 limits for orthopedic and orthodontic treatment were identified as 310–360 ng/mL and 258–302 ng/mL, respectively.^57^ This finding can be further explored to outline the cut-off values for various orthodontic treatment types in clinical orthodontic setups. The treatment window for any skeletal modulation was identified by the time when the individual reached maturity in the same cervical stage, as in early or late maturers.^53^ This concept can be further explored with respect to the various body types and body weight.

The meta-analysis also studied sex-related difference in serum IGF-1 levels. Individual female data showed a significant rise in serum IGF-1 from CVMI 2 to stages CVMI 3, 4 and 5, while males showed a significant rise from CVMI 2 to CVMI 3 and 4. Other studies are also in agreement and show peak IGF-1 in males at the CS4 stage (corresponding to CVMI staging),^21^,^22^,^44^,^49^ compared to females who peak at CS3 followed by a decline in levels.^21^,^22^,^44^,^46^,^49^,^55^ The delayed and sustained puberty in males occurs due to a combination of growth hormone secretion mediated by IGF-1 production and lower estrogen levels.^81^ The early pubertal peak in females can be explained based on the role of DHEAS in stimulating IGF-1 and enhancing estrogen production.^82^ Thus, DHEAS shows an earlier peak in females at CS3 than in males at CS4.^44^ This difference of IGF-1 levels between males and females also influences osteocalcin levels, which shows a statistically significant correlation with IGF-1 across all CVMI stages (*P*<0.01) in males and across CVMI 3–6 in females.^22^

The remaining studies in other biofluids, including GCF and saliva, were not included in the meta-analysis, but they are extremely important to review for the outcomes and limitations of the current literature. These biofluids have advantages of non-invasive and repeated collection. The comprehensive review of biomarkers in all biofluids may aid planning of future studies to generate a higher level of evidence for the most potent mediator in an opportune medium using a robust methodology.

Gingival crevicular fluid (GCF) has been explored sufficiently in orthodontic tooth movement,^16^,^17^ but its role in assessment of skeletal maturation markers is promising, yet not explored sufficiently. The current review shows a significantly higher ALP level in GCF at pubertal stage compared with the pre- or post-pubertal stage.^25^,^40^,^59^,^60^ The peak at the pubertal stage can be explained based on the role of ALP in skeletal bone mineralization, growth, and remodeling. Previous literature supports increased levels of serum ALP during pubertal growth in patients undergoing tooth movement.^83^ Serum ALP levels may influence GCF ALP levels,^25^ and association of local variables like dental eruption status affecting serum ALP still needs further exploration. The ALP levels in GCF can be measured both as absolute and normalized (relative to the total protein content). Of these, normalized ALP is shown to be more accurate than absolute ALP levels for growth markers in the current review.^59^,^60^

Other markers like vitamin D binding protein (DBP) and serotransferrin (TF) also show a higher GCF percentage in pubertal (CS3, 4) compared to non-pubertal stage.^24^,^45^ However, a normal range for each biomarker is required to be established for each cervical stage.

Similar to the ALP levels in GCF, salivary ALP activity was increased in the early pre-pubertal stage (CS1 compared with CS2), followed by peak salivary protein concentration in CS3 and CS5.^27^ However, these results slightly contradict previous literature which reported highest salivary ALP levels in pubertal spurt using MP3 staging, cervical vertebral maturation staging, or physical maturation showing a hormonal surge.^68^,^69^,^84^ The difference can be attributed to studying normalized ALP rather than absolute ALP levels in the current review. The only limitation in detecting ALP in saliva is that its level in saliva is 4–5 times less than in plasma.^85^ Hence, for saliva, highly sensitive high-throughput techniques are required to detect minute quantities of biomarkers, but these are costly and not available routinely.

Another sensitive and specific marker for bone formation is bone-specific alkaline phosphatase (BALP) which has been investigated previously for changes in bone volume and density corresponding to age or stages of sexual development and during orthodontic tooth movement.^86^,^87^ But it has not yet been explored for skeletal maturation in both saliva and serum. The current review brought forth one study that mentioned a regression equation for predicting pubertal onset using salivary BALP levels along with chronological age and body mass index (BMI) percentile.^23^ Their findings were supported by a previous study that showed serum BALP to peak at puberty,^88^ and thus it can be further explored for variation in saliva for predicting pubertal onset.

Higher salivary IGF-1 at high velocity of skeletal growth, or pubertal peak in QCVM II stage,^52^ is also shown in the current review. This finding is similar to IGF-1 levels in serum and to another study depicting salivary IGF-1 peak (6.15±1.04 pg/mL) at puberty.^37^,^38^,^43^,^89^ According to previous literature, the factors influencing levels of free circulating IGF-1 in saliva are body mass index (BMI) and malnutrition status,^90^ which may be further explored as contributing factors to skeletal maturation status.

The current review has discussed various biomarkers for skeletal maturation, and has brought forth many novel and interesting findings. Although only four studies on serum IGF-1 qualified for meta-analysis, it highlights the need for standardized robust methodology and assessment criteria for biomarker studies in skeletal maturation. Some areas that require further exploration include a need for biomarker cut-off levels for each cervical stage, studying the ratios of serum GH/IGF-1 and IGF-1/ IGFBP-3 rather than absolute IGF-1 levels, and investigating salivary BALP as a very sensitive predictor of pubertal onset along with age and BMI percentile.

### Study Limitations

1. Heterogeneity in the biomarker category, sample selection, method of analysis, and observation times. Lack of standardized protocol for sample collection timing, technique, transfer, and storage.
2. The difference in sensitivity in assays, laboratories, and populations potentially interferes with consistency in results and conclusions.

## CONCLUSION

1. Meta-analysis showed a significant difference in serum IGF-1 levels between CS3, CS4, and CS5 compared to CS2 in a combined sample of males and females. However, the interpretation of the association should be made with caution due to the heterogeneity of the original studies.
2. We found a statistically significant rise in biomarkers in GCF (DBP, TF, and ALP), saliva (ALP, IGF-1, and BALP), and blood and serum (IGF-1, IGF-1/IGFBP-3 ratio, osteocalcin, BALP) at the pubertal stage (CS3, CS4) compared with pre-pubertal (CS1, CS2) and post-pubertal stages (CS5, CS6).
3. Metabolites (glucose, mannose, pyroglutamic acid, glycerol, glyceric acid) show differences among pre-pubertal, pubertal, and post-pubertal stages of skeletal maturation. Further studies are required for confirmation.

### Further Recommendations

1. Future studies must consider heterogeneity in studies related to ethnicity, sample size, sexual dimorphism, and early or late maturity.
2. Saliva, GCF, and urine can be explored as non-invasive biofluids for marker assessment.
3. All body fluids must be simultaneously studied to draw biological associations and correlations with the pre-identified skeletal maturation index.
4. Metabolites may be explored further for association with skeletal, dental, and chronological age.
5. There is a need to identify and develop a robust skeletal maturity biomarker as a chairside, sensitive, reliable tool to aid clinical decision-making and choice of orthodontic treatment alternatives.

## Supplementary Information



## Abbreviations

ALP: alkaline phosphatase
BALP: bone-specific alkaline phosphatase
CS: cervical maturation stage(s)
CVM: cervical vertebral maturation
CVMI: cervical vertebral maturation index
DBP: vitamin-D binding protein
DHEAS: dehydroepiandrosterone sulfate
ELISA: enzyme-linked immunosorbent assay
GCF: gingival crevicular fluid
GH: growth hormone
IGF-1: insulin-like growth factor
MP3: middle phalanx of the third finger
PHV: peak height velocity
PTHrP: parathormone-related protein
QCVM: quantitative cervical vertebral maturation
QUADAS-2: Quality Assessment of Diagnostic Accuracy Studies
ROB: risk of bias
SME: skeletal maturity evaluation
SMI: skeletal maturity indicator
TF: serotransferrin
