# Supplementary material for: Biomarkers in Body Fluids as Indicators of Skeletal Maturity: A Systematic Review and Meta-analysis
Source: Rambam Maimonides Med J. 2023 Aug 30;14(4):e0021. doi: 10.5041/RMMJ.10506 (PMC10619988; doi:10.5041/RMMJ.10506)
Supplement: Supplementary file 1 [file rmmj-14-4-e0021-Supplement.pdf]

SUPPLEMENTARY MATERIAL

This appendix has been provided by the authors for the benefit of readers

Supplement to Biomarkers in Body Fluids as Indicators of Skeletal Maturity: A Systematic Review and Meta-analysis

Kapoor P, Balachandran R, Chowdhry A, Perinetti G, Kharbanda OP. Biomarkers in Body Fluids as Indicators of Skeletal Maturity: A Systematic Review and Meta-analysis. Rambam Maimonides Med J 2023;14 (4):e0021. Review. doi:10.5041/RMMJ.10506. Epub 2023 Aug 30.

Supplementary Table 1. Search Strategy Applied in Different Databases.

Database	Search Strategy
PubMed	(puberty OR pubertal OR skeletal maturation OR skeletal maturity OR skeletal development OR bone age OR skeletal age OR facial growth OR mandibular growth or maxillary growth OR growth phase OR growth peak OR spurt OR height peak) AND (molecule OR molecules OR enzyme OR enzymes OR factor OR factors OR marker OR markers OR biomarker OR biomarkers OR hormone OR hormones) AND (GCF OR crevicular OR saliva OR salivary OR serum OR blood) AND (cervical vertebral maturation OR cervical stage OR cervical maturation OR CVMI OR CVM OR MP3 OR third finger middle phalanx OR MPM OR Fishman OR Greulich Pyle OR Greulich-Pyle OR Lamparski OR Hassel Farman OR hand wrist OR hand-and-wrist OR standing height)
Scopus	(("skeletal maturation" OR "skeletal age" AND (gcf OR crevicular OR saliva OR salivary OR serum OR blood) AND (orthodontics) AND (biomarkers))
Web of Science	TS=((puberty OR pubertal OR "skeletal maturation" OR "skeletal maturity" OR "skeletal development" OR "bone age" OR "skeletal age" OR "facial growth" OR "mandibular growth" OR "maxillary growth" OR "growth phase" OR "growth peak" OR spurt OR "height peak") AND (molecule OR molecules OR enzyme OR enzymes OR factor OR factors OR marker OR markers OR biomarker OR biomarkers OR hormone OR hormones) AND (GCF OR crevicular OR saliva OR salivary OR serum OR blood) AND ("cervical vertebral maturation" OR "cervical stage" OR "cervical maturation" OR CVMI OR CVM OR MP3 OR "third finger middle phalanx" OR MPM OR Fishman OR Greulich Pyle OR Greulich-Pyle OR Lamparski OR Hassel-Farman OR hand wrist OR hand-and-wrist OR "standing height"))

Database	Search Strategy
Embase	('puberty'/exp OR puberty OR pubertal OR 'skeletal maturation'/exp OR 'skeletal maturation' OR 'skeletal maturity'/exp OR 'skeletal maturity' OR 'skeletal development'/exp OR 'skeletal development' OR 'bone age'/exp OR 'bone age' OR 'skeletal age'/exp OR 'skeletal age' OR 'facial growth'/exp OR 'facial growth' OR 'mandibular growth' OR 'maxillary growth'/exp OR 'maxillary growth' OR 'growth phase'/exp OR 'growth phase' OR 'growth peak' OR spurt OR 'height peak') AND ('molecule'/exp OR molecule OR 'molecules'/exp OR molecules OR 'enzyme'/exp OR enzyme OR 'enzymes'/exp OR enzymes OR factor OR factors OR 'marker'/exp OR marker OR markers OR 'biomarker'/exp OR biomarker OR 'biomarkers'/exp OR biomarkers OR 'hormone'/exp OR hormone OR 'hormones'/exp OR hormones) AND (gcf OR crevicular OR 'saliva'/exp OR saliva OR salivary OR 'serum'/exp OR serum OR 'blood'/exp OR blood) AND ((('cervical vertebral maturation' OR 'cervical stage' OR 'cervical maturation' OR cvmi OR cvm OR mp3 OR 'third finger middle phalanx' OR mpm OR fishman OR greulich) AND pyle OR 'greulichpyle' OR lamparski OR 'hasselfarman' OR 'hand'/exp OR hand) AND ('wrist'/exp OR wrist) OR 'hand and wrist' OR 'standing height'/exp OR 'standing height')
Directory of Open Access Journals (DOAJ)	biomarkers AND orthodontics AND skeletal maturation

See next pages for Supplemental Table 2, and Supplemental Figures 1 and 2.

Supplementary Table 2. Inclusion and Exclusion Criteria for Studies.

Inclusion Criteria	Exclusion Criteria
All original studies on humans including clinical trials, prospective or retrospective cohort studies, studies mentioning on both biomarkers and skeletal maturation, studies that compare biomarkers with radiographic assessment of skeletal maturation	In-vitro studies, animal studies, studies on biomarkers but not on skeletal maturation, studies on skeletal maturation but not on biomarkers, studies with no radiographic comparator, studies in unhealthy patients or those undergoing treatment, case reports, and reviews/opinions

See next pages for Supplemental Figures 1 and 2.

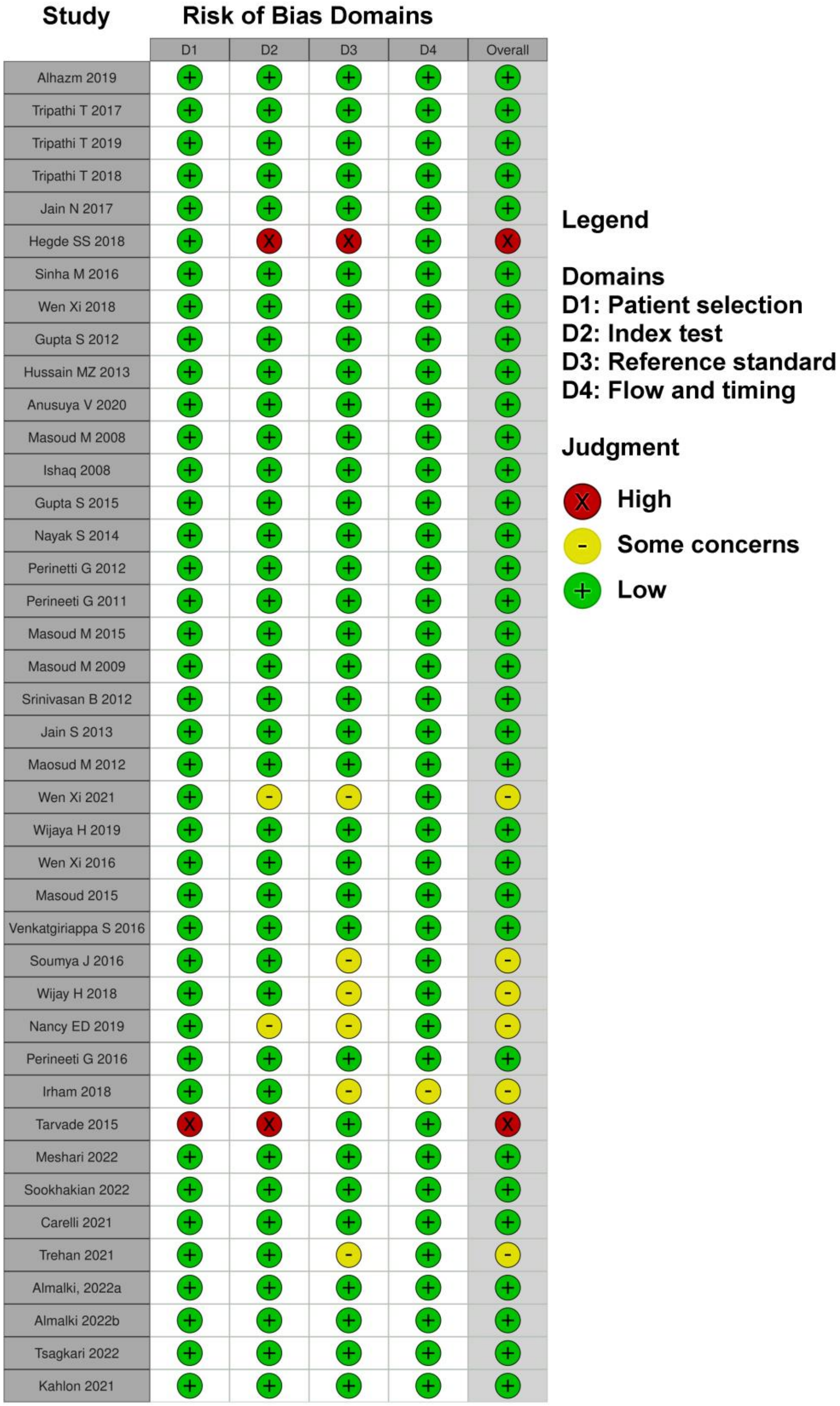

Supplementary Figure 1. Quality Assessment by QUADAS-2 Tool.

Quality assessment of 41 total eligible studies, and exclusion of 6 studies on the basis of an unclear risk of bias; high risk of bias in two studies.

A)

Study or Subgroup	CVMI3 Mean	CVMI3 SD	CVMI3 Total	CVMI4 Mean	CVMI4 SD	CVMI4 Total	Weight	Mean Difference IV, Random, 95% CI
Anushya et al. 2020	332.31	16.27	7	538.32	32.31	7	21.9%	−206.01 [−232.81, −179.21]
Ishaq et al. 2012	463	164	10	893	171	10	15.6%	−430.00 [−576.85, −283.15]
Jain et al. 2013	318	17.4	15	352	17.7	15	22.1%	−34.00 [−46.56, −21.44]
Kahlon et al. 2021	372.45	48.22	20	323.6	72.04	20	21.6%	48.85 [10.86, 86.84]
Tripati et al. 2017	384.62	139.84	13	528.04	114.15	14	18.8%	−143.42 [−240.14, −46.70]
**Total (95% CI)**			**65**			**66**	**100.0%**	**−136.19 [−242.91, −29.47]**

Heterogeneity: Tau² = 13359.19; Chi² = 189.22, df = 4 (P < 0.00001); I² = 98%
Test for overall effect: Z = 2.50 (P = 0.01)

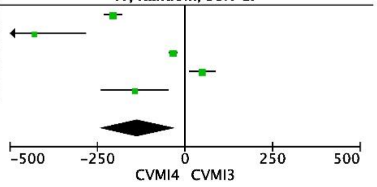

Study or Subgroup	CVMI5 Mean	CVMI5 SD	CVMI5 Total	CVMI3 Mean	CVMI3 SD	CVMI3 Total	Weight	Mean Difference IV, Random, 95% CI
Anushya et al. 2020	212.76	4.01	7	332.31	16.27	7	25.8%	−119.55 [−131.96, −107.14]
Ishaq et al. 2012	681	122	8	463	164	10	11.0%	218.00 [85.79, 350.21]
Jain et al. 2013	279	10.8	15	318	17.4	15	25.9%	−39.00 [−49.36, −28.64]
Kahlon et al. 2021	284.25	69.88	20	372.45	48.22	20	23.6%	−88.20 [−125.41, −50.99]
Tripati et al. 2017	384.17	132.13	12	384.62	139.84	13	13.7%	−0.45 [−107.07, 106.17]
**Total (95% CI)**			**62**			**65**	**100.0%**	**−37.95 [−95.34, 19.44]**

Heterogeneity: Tau² = 3279.81; Chi² = 116.23, df = 4 (P < 0.00001); I² = 97%
Test for overall effect: Z = 1.30 (P = 0.19)

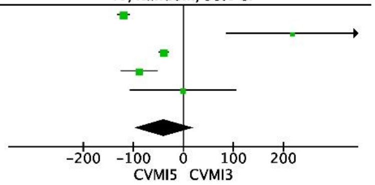

Study or Subgroup	CVMI5 Mean	CVMI5 SD	CVMI5 Total	CVMI4 Mean	CVMI4 SD	CVMI4 Total	Weight	Mean Difference IV, Random, 95% CI
Anushya et al. 2020	212.76	4.01	7	538.32	32.31	7	21.1%	−325.56 [−349.68, −301.44]
Ishaq et al. 2012	681	122	8	893	171	10	17.6%	−212.00 [−347.57, −76.43]
Jain et al. 2013	279	10.8	15	352	17.7	15	21.2%	−73.00 [−83.49, −62.51]
Kahlon et al. 2021	284.25	69.88	20	323.6	72.04	20	20.8%	−39.35 [−83.34, 4.64]
Tripati et al. 2017	384.17	132.13	12	528.04	114.15	14	19.3%	−143.87 [−239.60, −48.14]
**Total (95% CI)**			**62**			**66**	**100.0%**	**−157.46 [−296.03, −18.90]**

Heterogeneity: Tau² = 23542.84; Chi² = 367.16, df = 4 (P < 0.00001); I² = 99%
Test for overall effect: Z = 2.23 (P = 0.03)

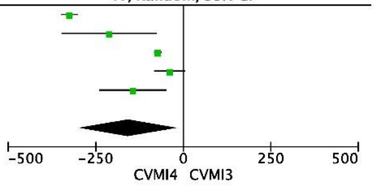

B)

Study or Subgroup	CVMI3 Mean	CVMI3 SD	CVMI3 Total	CVMI4 Mean	CVMI4 SD	CVMI4 Total	Weight	Mean Difference IV, Random, 95% CI
Anushya et al. 2020	460.38	13.49	7	228.33	8.96	7	17.8%	232.05 [220.05, 244.05]
Ishaq et al. 2012	582	175	9	794	217	14	13.0%	−212.00 [−373.22, −50.78]
Jain et al. 2017	403.3	12.3	15	332.4	12.8	15	17.9%	70.90 [61.92, 79.88]
Kahlon et al. 2021	353.4	43.11	20	313.65	72.08	20	17.5%	39.75 [2.94, 76.56]
Sinha et al. 2016	329.49	22.17	12	410.63	11.92	12	17.8%	−81.14 [−95.38, −66.90]
Tripati et al. 2017	527.5	87.94	14	507.88	153.1	14	15.9%	19.62 [−72.87, 112.11]
**Total (95% CI)**			**77**			**82**	**100.0%**	**22.09 [−89.49, 133.67]**

Heterogeneity: Tau² = 18129.84; Chi² = 1130.07, df = 5 (P < 0.00001); I² = 100%
Test for overall effect: Z = 0.39 (P = 0.70)

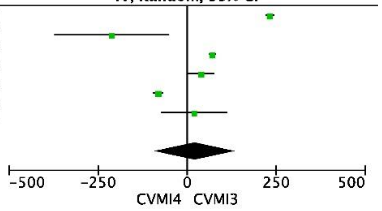

Study or Subgroup	CVMI5 Mean	CVMI5 SD	CVMI5 Total	CVMI3 Mean	CVMI3 SD	CVMI3 Total	Weight	Mean Difference IV, Random, 95% CI
Anushya et al. 2020	206.31	6.77	7	460.38	13.49	7	18.5%	−254.07 [−265.25, −242.89]
Ishaq et al. 2012	544	207	7	582	175	9	9.6%	−38.00 [−229.28, 153.28]
Jain et al. 2017	294.5	10.9	15	403.3	12.3	15	18.5%	−108.80 [−117.12, −100.48]
Kahlon et al. 2021	265.1	68.44	20	353.4	43.11	20	18.0%	−88.30 [−123.75, −52.85]
Sinha et al. 2016	303.12	7.63	12	329.49	22.17	12	18.5%	−26.37 [−39.64, −13.10]
Tripati et al. 2017	382.75	70.94	12	527.587	87.94	14	16.9%	−144.84 [−205.94, −83.74]
**Total (95% CI)**			**73**			**77**	**100.0%**	**−116.08 [−201.24, −30.91]**

Heterogeneity: Tau² = 10174.25; Chi² = 738.25, df = 5 (P < 0.00001); I² = 99%
Test for overall effect: Z = 2.67 (P = 0.008)

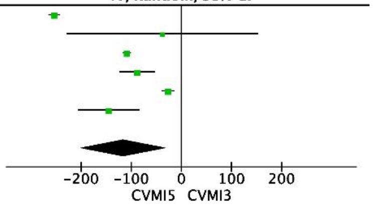

Study or Subgroup	CVMI5 Mean	CVMI5 SD	CVMI5 Total	CVMI4 Mean	CVMI4 SD	CVMI4 Total	Weight	Mean Difference IV, Random, 95% CI
Anushya et al. 2020	206.31	6.77	7	228.33	8.96	7	22.2%	−22.02 [−30.34, −13.70]
Ishaq et al. 2012	544	207	7	794	217	14	4.0%	−250.00 [−440.88, −59.12]
Jain et al. 2017	294.5	10.9	15	332.4	12.8	15	22.2%	−37.90 [−46.41, −29.39]
Kahlon et al. 2021	265.1	68.44	20	313.65	72.08	20	18.1%	−48.55 [−92.11, −4.99]
Sinha et al. 2016	303.12	7.63	12	410.63	11.92	12	22.2%	−107.51 [−115.52, −99.50]
Tripati et al. 2017	382.75	70.94	12	507.88	153.1	14	11.2%	−125.13 [−214.81, −35.45]
**Total (95% CI)**			**73**			**82**	**100.0%**	**−70.11 [−112.49, −27.72]**

Heterogeneity: Tau² = 2087.38; Chi² = 246.34, df = 5 (P < 0.00001); I² = 98%
Test for overall effect: Z = 3.24 (P = 0.001)

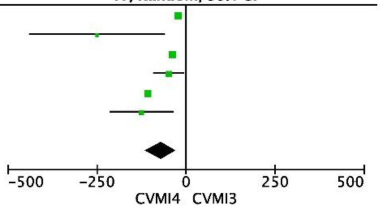

Supplementary Figure 2. Comparison of Serum IGF-1 Levels Between CVMI 3, 4, 5 in Males (A) and Females (B). The CVMI staging is also referred to as cervical stages (CS).

CVMI, cervical vertebral maturation index.
